# Exploring Undergraduate Students’ Perspectives on Endodontic Treatment: A Cross-Sectional Study in Makkah

**DOI:** 10.7759/cureus.62885

**Published:** 2024-06-22

**Authors:** Abdelrahman M Alhilou

**Affiliations:** 1 Endodontics, Umm Al-Qura University, Makkah, SAU

**Keywords:** education, endodontics, students, undergraduate, perceptions

## Abstract

Background

Students at two universities in Saudi Arabia found endodontics or root canal treatment (RCT) difficult due to challenges in the procedures. Until now, there has been no evidence that Umm Al-Qura University students face difficulties when performing RCT. Therefore, this study aims to explore students' perceptions of the critical steps in RCT.

Methods

A survey was conducted among 146 dental students at Umm Al-Qura University, Makkah, Saudi Arabia to assess their perceptions and challenges during RCT. Demographic information was collected in the first section, while the second section focused on identifying difficulties encountered during endodontic treatment stages. All participants provided signed electronic consent, and the study was approved by the university’s institutional review board. Chi-squared tests were used to analyze the results.

Results

In a survey of 123 students, 94 (76.2%) found the endodontic specialty acceptable compared to other dental specialties (P < 0.001). Eighty-eight (71.5%) found RCT on the molars difficult (P < 0.001). Most students, 104 (84.6%), chose to have more clinical training to improve their clinical endodontic skills, and 77 (62.6%) chose to increase the number of instructors per student in the clinic (P < 0.05). Difficulties in performing RCT on premolars and during root canal obturation were statistically correlated with the academic year of the student (P < 0.05).

Conclusion

Dental students often struggle when performing RCT on molars. Some difficulties during RCT are correlated with the academic year of the student. Finally, from the students’ perspective, improving endodontic skills involves providing better clinical training opportunities and increasing instructor-to-student ratios.

## Introduction

Endodontic treatment can be difficult for dental practitioners due to its complexity. More people want to keep their natural teeth, which means there will likely be a higher demand for endodontic procedures in the future [1]. Among different dental specialties, endodontics is one of the toughest for students to learn because there are many challenges involved. These challenges include communicating with patients, managing emergencies, using different tools and materials, addressing root canal issues, and avoiding errors. These challenges can cause stress for students and may lead to poor results for patients [2]. In order to effectively overcome these challenges, it is essential to provide students with thorough training and education. The quality of education a student receives is often reflected in their confidence and proficiency in a particular field [3]. Therefore, it is crucial to invest in strong educational resources to help build the confidence and competence of students.

The endodontic course for dental students at Umm Al-Qura University in Makkah, Saudi Arabia is initiated in the fourth year of a six-year Bachelor of Dental Surgery (BDS) program. The preclinical course (in the fourth year) is designed to cover endodontic topics through a combination of lectures and practical applications. During the practical sessions, students are required to apply their knowledge to artificial teeth that are mounted on a dental simulator mannequin head. This hands-on approach enables students to gain a comprehensive understanding of endodontic practices and prepares them for clinical work. In the fifth and sixth years of their academic progression, students are required to undertake an integrated course that encompasses five disciplines, namely periodontics, operative dentistry, endodontics, fixed prosthodontics, and removable prosthodontics. The course is divided into two main components: a theoretical aspect delivered through self-directed learning sessions, and a clinical component, which encompasses practical clinical sessions allowing students to apply the knowledge they have gained. It is expected that upon completion of the courses, students will be able to proficiently perform various dental procedures and possess a comprehensive understanding of the underlying principles and techniques. This will enable them to function effectively in their future careers as dentists, providing high-quality dental care to their patients.

Many dental students at two universities in Saudi Arabia find endodontics to be a challenging branch of dentistry. This is attributed to a number of factors, including the complexity of managing emergency situations and pain, apprehension regarding potential mishaps during the procedure, and anatomical variations related to the teeth being treated [4,5]. However, it is unknown if students at Umm Al Qura University encounter challenges with root canal treatment (RCT). As such, the aim of this study is to provide insight into the students’ perceptions of the most critical steps involved in RCT at Umm Al-Qura University.

## Materials and methods

Study design

A survey was conducted among all 146 undergraduate dental students enrolled in the fifth year, sixth year, and internship year at the Faculty of Dentistry at Umm Al-Qura University, Makkah, Saudi Arabia during the academic year 2023-2024. The participants consisted of 51 fifth-year students, 43 sixth-year students, and 52 internship-year students, including both male and female dental students. The survey comprised 23 electronically distributed questions, which were divided into two sections. The first section aimed at collecting demographic information (age, gender, and student academic year), while the second section focused on assessing the students’ perceptions and the challenges encountered during RCT. The survey also evaluated the students’ opinions on improving their clinical skills and their perceptions about their career intentions regarding endodontic practice after graduation. The survey questions are presented in a table within the appendix.

Ethical considerations

This study was granted ethical approval from the Umm Al-Qura University Institutional Review Board (approval number HAPO-02-K-012-2023-10-1802). All participants who agreed to participate in the survey provided signed electronic consent before taking the survey. The survey utilized was adapted from previous studies to suit the current research objectives [6].

Statistical analysis

Chi-squared tests were used to compare the survey responses in percentage, reflecting students’ perceptions of each question. Furthermore, Pearson correlation tests were used to identify the relationship between the difficulties encountered during endodontic treatment, by academic year, and by gender. The significance level was established as P < 0.05. Statistical analysis was performed using IBM SPSS Statistics for Windows, Version 27.0 (Released 2020; IBM Corp., Armonk, NY, USA).

## Results

Demographic data

Of the 146 students who were invited to participate in the survey, a total of 123 responded, resulting in a response rate of 84.24%. Among the participants, there were 77 (62.6%) females and 46 (37.4%) males. The majority of the respondents were from the fifth year, followed by the sixth year and the internship year, with 66 (53.7%), 35 (28.5%), and 22 (17.9%) students, respectively. Based on the data, 82 (66.7%) of students are aged between 21 and 23 years, 39 (31.7%) between 23 and 26 years, and only two (1.6%) between 18 and 20 years.

The perceptions of students

**Figure 1:**
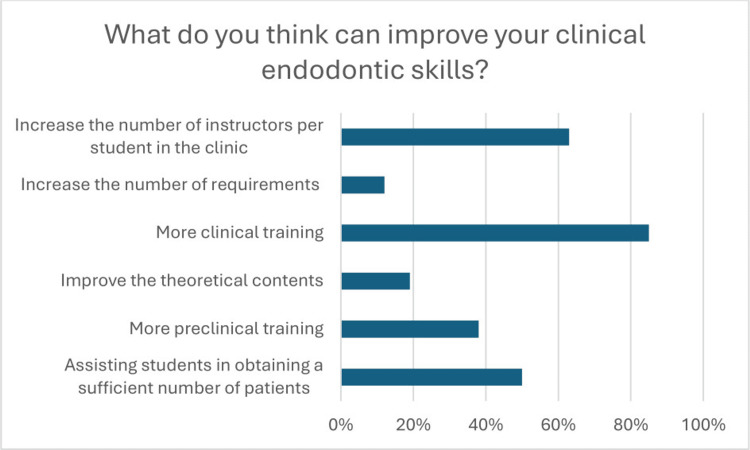
Percentage of students who choose different options on what can improve students’ clinical endodontic skills

Most students, 94 (76.2%), find the endodontic specialty acceptable compared to other dental specialties (P < 0.001). The majority of respondents, 88 (71.5%), found RCT on molars to be difficult (P < 0.001), while it was found to be acceptable (P < 0.001) and easy (P < 0.001) for premolars and anteriors, respectively. When asked about options that can improve students’ clinical endodontic skills, the majority (104, 84.6%) chose to have more clinical training (P < 0.001). Additionally, 77 (62.6%) of the students chose to increase the number of instructors per student in the clinic (P = 0.005). These were the two most common answers, as shown in Figure 1.

All the rest of the questions with students’ answers regarding endodontic treatment difficulties are presented in Table 1.

**Table 1:** Number (percentage) of students who responded with “Yes” or “No” for each question, along with their corresponding P-values and chi-square values

Questions	Answers		P-Value	Chi-square value
	Yes	No		
Is there any difficulty in performing anesthesia?	23 (18.7%)	100 (81.3%)	P < 0.001	48.203
Is there any difficulty during the rubber dam application?	21 (17.1%)	102 (82.9%)	P < 0.001	53.341
Is there any difficulty while taking radiographs?	65 (52.8%)	58 (47.2%)	P = 0.528	0.398
Is there any difficulty during the endodontic access cavity?	39 (31.7%)	84 (68.3%)	P < 0.001	16.463
Is it difficult to check and remove the pulp chamber roof?	45 (36.6%)	78 (63.4%)	P = 0.003	8.854
Is it difficult to locate and detect orifices?	71 (57.7%)	52 (42.3%)	P = 0.087	2.935
Is there any difficulty during working length determination?	56 (45.5%)	67 (54.5%)	P = 0.321	0.984
Do you use an apex locator for working length determination?	109 (88.6%)	14 (11.4%)	P < 0.001	73.374
Is there any difficulty during working length determination using the apex locator?	43 (35.0%)	80 (65.0%)	P < 0.001	11.13
Is there any difficulty during root canal chemomechanical instrumentation?	24 (19.5%)	99 (80.5%)	P < 0.001	45.732
Is there any difficulty during root canal obturation?	58 (47.2%)	65 (52.8%)	P = 0.528	0.398
Is there any difficulty when applying cotton pellets before temporization?	13 (10.6%)	110 (89.4%)	P < 0.001	76.496
Is there any difficulty when applying temporary fillings?	12 (9.8%)	111 (90.2%)	P < 0.001	79.683
Are you worried about making mistakes like perforating the tooth, breaking instruments inside the canals, or forcing irrigation out of the root canals?	109 (88.6%)	14 (11.4%)	P < 0.001	73.374
Can these difficulties affect your decision to choose endodontics as a future specialty?	64 (52%)	59 (48%)	P = 0.652	0.203

Significant relationship

**Figure 2:**
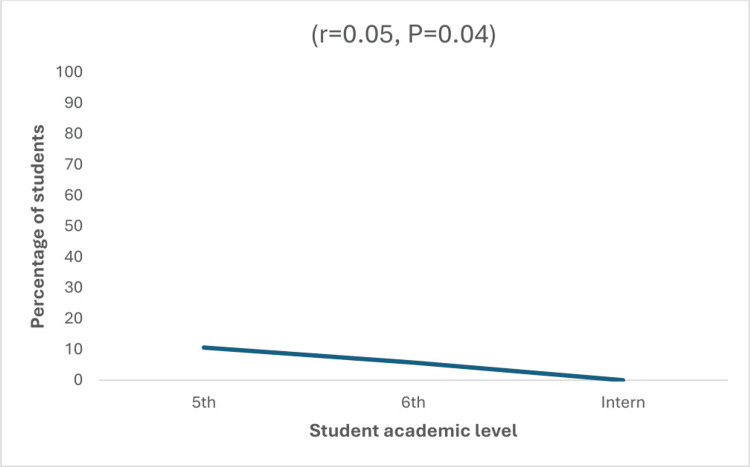
Correlation between the academic year (level) of the students and the percentage of students who found doing RCT on a premolar difficult 5th, fifth academic year; 6th, sixth academic year; intern, internship education year; r, Pearson; RCT, root canal treatment

There was a statistically significant correlation between students’ academic year and the percentage of students who found RCT on premolars difficult (r = 0.051, P = 0.040). In other words, as students progressed in their academic years, the percentage of students who found RCT on premolars difficult decreased (Figure 2).

Moreover, a statistically significant correlation was found between students’ academic year and difficulties during root canal obturation, with root canal obturation difficulty reported higher among those in the fifth year compared to other years (r = 0.089, P = 0.021). The study also found a significant correlation between the academic year and students’ future choice of endodontics as a specialty, where a higher number of students, 33 (50%) in the fifth year, mentioned that difficulties encountered during endodontic treatment could affect their decision to choose endodontics as their future specialty (r = 0.106, P = 0.021). There was no significant correlation between students’ academic year and their age (P > 0.05).

## Discussion

Students have a variety of specialties to choose from in dentistry. However, based on the results, the majority of students have shown an inclination toward studying endodontics compared to other specialties. This trend is not new in Saudi Arabia, as previous studies have also reported similar results [7,8]. Several factors must be considered when deciding on a specific field of dentistry. These may include the complexity of diagnostic problems, the requirement for specialized skills or talents in a particular area of dentistry, and the intellectual demands of the field. Furthermore, one may also take into account any possible influence from family members who work in the same field [9].

According to the study’s findings, the majority of respondents found RCT on molars to be difficult. This finding is consistent with numerous studies that have shown that molar endodontics is the most challenging treatment for students [10-12]. Performing an RCT on molars can be challenging due to several factors, such as the complex anatomy of molar teeth, the presence of additional canals (such as the MB2 canal), difficulty in accessing and locating all canals, inadequate access openings, and the risk of missed canals. The level of training and experience of the student performing the RCT can also impact the outcome. Patient-related factors such as limited mouth opening, severe gag reflex, and behavioral management issues in anxious or uncooperative patients are other important relative factors [13-15].

The results of this study demonstrate that there is a significant correlation between the academic year of the students and the challenges they face while performing RCT on premolars as well as during root canal obturation. This correlation can be attributed to the fact that students develop their expertise over the years, making it easier for them to perform RCT with greater proficiency [14,15].

Although sixth-year students had less experience in endodontics than internship students, a lower percentage (28%) of sixth-year students found root canal obturation more difficult than internship students (45%). Additionally, a higher percentage of internship students (77%) reported that difficulties encountered during endodontic treatment could affect their decision to choose endodontics as their future specialty, compared to sixth-year students (40%). These findings seem to suggest that factors other than experience might potentially influence the perceived difficulty of certain procedures. For example, students in their sixth year might have recently received formal education and training in RCT, which could have focused on the fundamental principles and technical skills required, making it feel more manageable. On the other hand, internship students might have encountered a wider variety of cases and complexities, potentially leading to a heightened awareness of the potential challenges and intricacies involved in RCT. Additionally, the pressure and expectations of being in an internship role might seem to contribute to a perceived increase in difficulty for these students, despite their advanced experience [16].

The results of the questionnaire revealed that students believe that their clinical endodontic skills can be improved through increased clinical training and a higher number of instructors per student in the clinic. This information supports previous studies that have shown how important practical clinical experience and tailored instruction can be in improving students’ endodontic skills [17-19]. The impact of increased clinical training suggests that the chance to practice under supervision is essential for skill development. Moreover, having a higher number of instructors per student in the clinic can provide more personalized feedback and guidance, which is likely to contribute to improved skill acquisition [20]. These findings underline the significance of optimizing the learning environment to support the development of clinical endodontic skills among students.

One must not forget the limitations of this research. Since the study is based on the data collected from students via self-completed questionnaires, some sources of bias that are relevant to this type of assessment may occur, such as recall and response bias. Further, the research is cross-sectional, thus making it a “snapshot” of the sampled students and their perceptions at a given time. Lastly, confounding factors such as the learning mode or style can be considered a limitation that could affect the results.

## Conclusions

This study provides insights into the perceptions and difficulties encountered by dental students during their endodontic training. Within the limitations of the study, students in the fourth, fifth, and internship years at Umm Al-Qura University have shown an interest in endodontics as a specialty compared to other specialties. From students’ perspectives, performing RCT on anterior and premolars was manageable, but it was more challenging when working on molars. As students progress in their academic year, the difficulty of certain steps during RCT decreases, which can indicate improvement in endodontic skills over the years of education. Moreover, the students suggested providing an environment that facilitates learning, with more clinical training and instructors per student, to enhance their clinical endodontic skills. Addressing these difficulties and making suggestions may contribute to improving the proficiency of new students in the future.

## Appendices

**Table 2:** Survey questions and their corresponding answer options

Questions	Answers		
Age?	18-20	21-23	24-26
Gender?	Male	Female	
Student academic year?	5th year	6th year	Internship
What do you think about endodontics in terms of difficulty compared to other dental specialties?	Easy	Acceptable	Difficult
How difficult is doing root canal treatment (RCT) on anterior teeth?	Easy	Acceptable	Difficult
How difficult is doing RCT on premolars?	Easy	Acceptable	Difficult
How difficult is doing RCT on Molars?	Easy	Acceptable	Difficult
Is there any difficulty in performing anesthesia?	Yes	No	
Is there any difficulty during the rubber dam application?	Yes	No	
Is there any difficulty while taking radiographs?	Yes	No	
Is there any difficulty during endodontic access to the cavity?	Yes	No	
Is it difficult to check and remove the pulp chamber roof?	Yes	No	
Is it difficult to locate and detect orifices?	Yes	No	
Is there any difficulty during working length determination?	Yes	No	
Do you use an apex locator for working length determination?	Yes	No	
Is there any difficulty during working length determination using the apex locator?	Yes	No	
Is there any difficulty during root canal chemomechanical instrumentation?	Yes	No	
Is there any difficulty during root canal obturation?	Yes	No	
Is there any difficulty when applying cotton pellets before temporization?	Yes	No	
Is there any difficulty when applying temporary filling?	Yes	No	
Are you worried about making mistakes like perforating the tooth, breaking instruments inside the canals, or forcing irrigation out of the root canals?	Yes	No	
Can these difficulties affect your decision to choose endodontics as a future specialty?	Yes	No	
